# An introduction to biomarkers: applications to chronic kidney disease

**DOI:** 10.1007/s00467-007-0455-9

**Published:** 2007-03-30

**Authors:** Kevin V. Lemley

**Affiliations:** Division of Nephrology MS 40, Childrens Hospital Los Angeles, 4650 Sunset Blvd, Los Angeles, CA 90027 USA

**Keywords:** CKD, Surrogate endpoint, Cross validation, Proteomics, ROC curve

## Abstract

Diagnosis and management of chronic kidney disease (CKD) will be characterized in the future by an increasing use of biomarkers—quantitative indicators of biologic or pathologic processes that vary continuously with progression of the process. “Classical” biomarkers of CKD progression include quantitative proteinuria, the percentage of sclerotic glomeruli or fractional interstitial fibrosis. New candidate biomarkers (e.g., urinary proteomic patterns) are being developed based on both mechanistic and “shotgun” approaches. Validation of potential biomarkers in prospective studies as surrogate endpoints for hard clinical outcomes is often complicated by the long lag time to the ultimate clinical outcome (e.g., end-stage renal disease). The very dense data sets that result from shotgun approaches on small numbers of patients carry a significant risk of model overfitting, leading to spurious associations. New analytic methods can help to decrease this risk. It is likely that clinical practice will come to depend increasingly on multiplex (vector) biomarkers used in conjunction with risk markers in early diagnosis as well as to guide therapy.

## Introduction

Historically, kidney diseases have been described in terms of clinical observations supplemented by chemical measurements on blood or urine reflecting changing levels of organ function. Recently, there has been increasing interest in the use of biomarkers as clinical research tools in nephrology. This review will discuss some characteristics of biomarkers and illustrate some of their potential uses and limitations, particularly in the context of evaluation and treatment of chronic kidney disease (CKD). The use of biomarkers in acute kidney injury and transplantation will not be discussed.

The term biomarker has been in use since at least the 1970s, initially in clinical research on cancer and cardiovascular disease. More recently, biomarkers have become a topic of interest in the context of renal disease [1, 2]. As defined by a US National Institutes of Health (NIH) working group [3], a biomarker is a quantitative indicator of a definable biologic or pathologic process that may be used in diagnosis or to monitor therapy. The biomarker ideally varies continuously (usually monotonically) with the level of activity or degree of progression of the disease process. Frequently, biomarkers are measurable intermediary components of molecular or cellular pathways involved in the biologic process under consideration and as such represent a temporal slice through one of possibly many pathways that converge to define that process. The process itself may be disease (in which case the biomarker may be used in diagnosis or to assess efficacy of therapy) or treatment-related toxicity. The use of biomarkers in “development and evaluation of novel therapies” has gained regulatory significance with respect to NIH-sponsored clinical trials and US Food and Drug Administration (FDA) drug approval [3].

An ideal biomarker is noninvasive, allowing for repeated measurements. It should also be simple, quantitative, and accurate to measure; monotonically related to the biologic process under consideration; predictive of progression of that process (i.e., abnormal values in the marker precede the development of irreversible injury); and mechanistically meaningful in understanding the pathophysiology of the process. If the biologic process is considered as a trajectory through “biospace”, the biomarker is a numeric representation of the distance traveled along that trajectory (a metric). A biomarker need not be a chemical entity, such as the blood concentration of a particular metabolite: examples of nonchemical biomarkers include cyst size in polycystic kidney disease, renal pelvic diameter in prenatal ultrasounds, power-Doppler signal in renal ultrasound, and characteristic statistics representing 24-h ambulatory blood pressure recordings.

**Fig. 1:**
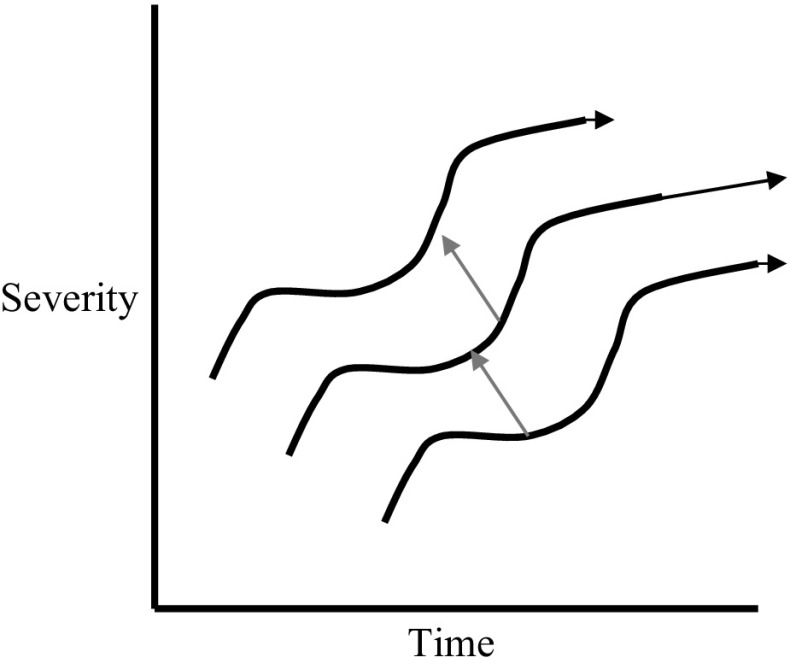
Different courses of disease progression represented as trajectories of a severity index versus time. The *tails* of the *short gray arrows* correspond to the same biomarker value (trajectory metric). The translation from one curve to another along these arrows is due to variation in some “modifying” parameter, such as a risk factor

A biomarker is different from a risk factor or susceptibility marker: A biomarker is a dynamic index of biologic activity. It has a tempo and trajectory. A risk factor may exist independently of the presence or absence of any renal injury. Smoking is an example of an environmental/behavioral risk factor. Examples of genetic risk factors include homozygous angiotensin converting enzyme (ACE) deletion genotype or certain α-adducin gene polymorphisms. These genetic risk factors may be responsible for a hypertensive milieu that deleteriously modifies the context of a renal disease, shifting the process from one trajectory to another “parallel” trajectory (Fig. 1). Although hypertension is often described as a risk factor for cardiovascular disease, as a dynamic process, it is better considered as a biomarker, one whose trajectory intersects with and influences other cardiovascular disease processes, such as atherosclerosis or cardiac hypertrophy.

There are many examples of biomarkers that have been applied to clinical problems other than CKD. For example, HIV viral load and CD4 cell counts are biomarkers for the pathologic process of HIV-induced immune suppression and may predict such clinical outcomes as opportunistic infection or death. Blood pressure and serum cholesterol concentration are biomarkers for the pathologic process of vascular injury/atherosclerosis and may predict cardiovascular and cerebrovascular events. Serum levels of C3 and C4 complement are biomarkers of pathologic processes involving complement consumption due to immune complex activation and may predict immune-mediated (inflammatory) tissue injury.

Surrogate endpoints are a subclass of biomarkers that are strongly associated with distant clinical events. They can reliably substitute for hard clinical endpoints in predicting clinical benefit, lack of benefit, or harm from a treatment [3]. In this context, “hard” implies that the endpoints are both unambiguous and usually final (irreversible), such as end-stage renal disease (ESRD) or death. The National Institute of Diabetes and Digestive and Kidney Diseases (NIDDK) of the NIH has encouraged the use of biomarkers, such as albuminuria, as surrogate endpoints to improve clinical trial efficiency and decrease the need for lengthy studies of slowly progressive diseases [4].

In addition to their use in clinical trials (for drug screening, patient preselection, or as surrogate endpoints), biomarkers can be used for tracking the natural history of disease in early studies. They will someday be used in clinical practice for the early detection of disease states or for monitoring treatment efficacy or toxicity. Biomarkers may extend, refine, or even eventually replace classical diagnostic labels: e.g., in renal pathology, biopsy gene expression may be used for molecular subtyping of focal segmental glomerular sclerosis (FSGS) and other nephropathies [5–8]. Proteomic biomarkers may be equally useful in molecular diagnostics, especially in settings in which significant posttranslational modification of proteins occurs, limiting the interpretation of gene expression arrays. The potential of metabolomics (the study of patterns of large numbers of small molecule metabolites) to supply biomarkers of CKD will likely unfold as appropriate analytical platforms and computational methods are developed to process the massive number of potentially relevant chemical species. Metabolomics may in fact supply the “fingerprint” of metabolic systems that most closely reflects the clinical phenotype. The metabolomic profile serves as a nexus connecting genomic and proteomic scaffolds. A forward-looking use of biomarkers is based on biomarker discovery, validation, and translation to clinical practice [1].

### Biomarker discovery

There are two principal roads to biomarker discovery: pathophysiologic investigations of specific disease pathways and “shotgun” approaches. Targeted pathophysiologic investigations, especially on animal models of disease, have been the predominant method of biomarker discovery in the past. The primary goal of these studies is an understanding of disease mechanisms; biomarker discovery is a secondary outcome. Shotgun approaches consist of exhaustive quantitative analyses of mRNA species or proteins in patient samples (tissue, blood, urine), often using “unsupervised” analytical methods to search for patterns in the resulting large data sets, with minimal constraints preset by the investigator. Of the shotgun approaches, urinary proteomics [9, 10] and tissue gene expression arrays [5, 7] are two of the most highly developed methods for finding potential biomarkers. Weaknesses of shotgun approaches include data overload, with the potential for overfitting of models [11], and a lack of immediate pathophysiologic insight. The risk of spurious associations is clear when one considers that there are more than 10^60^ ways of choosing 20 markers from a set of 10,000 possible markers. Strengths of shotgun approaches include the ability to rapidly screen huge numbers of possible associations and the increased likelihood of serendipitous discovery.

In general, the state of development of discovery and statistical methods in cancer research has been far advanced compared with that in nephrology. Gene expression arrays have been used to develop molecular portraits of various cancers with respect to their diagnostic class [12] or their response to therapy [13]. Serum proteomics has uncovered patterns associated with improved detection of ovarian cancer [14, 15] compared with the classic serologic marker of advanced ovarian cancer (CA125) used alone.

Data-mining strategies for the shotgun methods can be categorized as supervised, unsupervised, and semisupervised methods. With unsupervised methods, data is allowed to speak for itself, with clustering of similar individuals based only on some concept of distance in the data space. The number of clusters may be specified in advance or chosen so as to optimize the amount of variance explained per cluster. Hierarchical clustering of gene expression array data is a familiar example of an unsupervised method. In supervised methods, raw data are prepartitioned by a supervising variable, such as clinical outcome, before more unconstrained methods are applied. Semisupervised methods are machine-learning algorithms, in which some structural constraints are applied to data organization based on a (usually small) number of “labeled” cases (i.e., cases with known clinical outcomes), with the majority of data remaining unlabeled. This allows a more creatively ambiguous assignment in regions where classes overlap and thus enhances the stability of the overall classifications by optimizing the balance between imported (and presumably sometimes incorrect) knowledge and flexibility of the algorithm to find the best fit to the data.

### Biomarker validation

Validation of the performance of a new biomarker is the most problematic aspect of biomarker development. Biomarkers are validated either via their predictive associations with important hard clinical outcomes, or their agreement with diagnoses arrived at by gold-standard methods. The most compelling mechanistic reasoning cannot substitute for empirical verification of the predictive association of the biomarker with a clinical outcome. There are numerous examples of therapies that have reliably resulted in a credible surrogate outcome but which were not followed by the ultimate “hard” clinical outcome originally thought to be clearly associated with that surrogate [16]. For example, although treatment with atenolol reliably lowers systemic blood pressure (as reflected in brachial artery pressures, the surrogate endpoint), it has not been found to lower the incidence of cardiovascular events (hard clinical endpoint) as much as other antihypertensive medications having comparable effects on blood pressure [17]. An explanation of this discrepancy has recently been offered based on the finding that atenolol does not seem to lower the more pathogenetically relevant aortic blood pressures to the same degree as other treatments having the same effect on brachial artery pressures [18]. This is thus an example of a problem of adequacy of the surrogate. So, “considerable skepticism about conventional wisdom should accompany the adoption of biomarkers as a substitute for outcomes” [3].

**Fig. 2:**
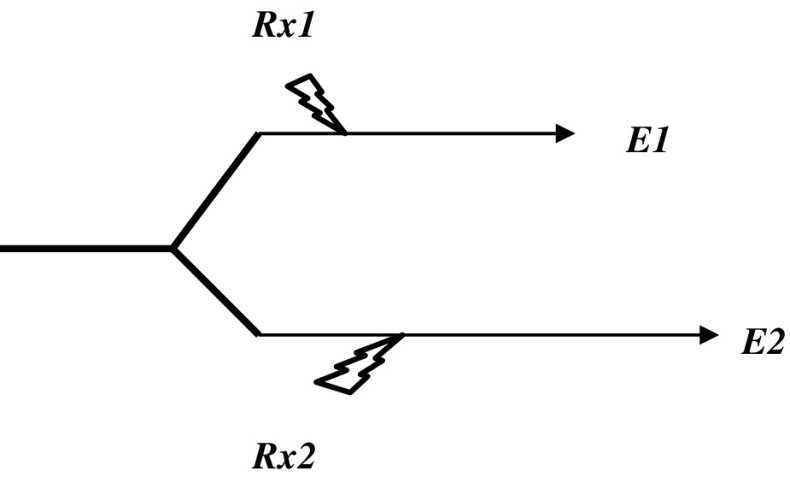
Although in the absence of treatment the surrogate endpoint *E*_*1*_ always precedes hard clinical endpoint *E*_*2*_, their relationship is an epiphenomenon. Treatment *Rx1* alters the surrogate without any clinical effect, and treatment *Rx2* has a clinical effect without an effect on the surrogate. Redrawn/modified from [16]

Even if there is a significant association of the surrogate biomarker to the clinical endpoint, it may not be obvious how rigidly this association stands up to perturbations [19]. A biomarker may be a perfect surrogate of the clinical endpoint in the untreated state, but the relationship between the surrogate and clinical endpoint may change significantly under the effects of treatment. In the above-mentioned case, for example, natural history studies have clearly shown measured brachial artery pressures to predict clinical cardiovascular events. It is in the therapeutic setting that the relationship is apparently altered. Pivotal questions are: What percentage of a treatment effect can be accounted for by treatment-induced changes in the biomarker? How well do changes in the surrogate endpoint under treatment capture the expected changes in the clinical endpoint? Put simply, a 50% normalization in the surrogate parameter, for example, should predict about a 50% improvement in the ultimate clinical outcome; that is, a high proportion of the treatment effect should be explained by treatment-induced changes in the biomarker. Unless the surrogate biomarker is the sole proximate cause of the clinical endpoint, however, this may not be the case. The pathways to a surrogate biomarker and the clinical endpoint may diverge before the point at which the therapy is targeted [16] (Fig. 2). Similarly, to compare two treatments using a surrogate endpoint, it is necessary that the correlation between treatment-induced changes in the surrogate and the hard outcome be equivalent under each treatment [19]. This is more likely to be the case if the treatments act by similar mechanisms, which, of course, may tend to diminish the inherent interest of the comparison. In addition, even a closely correlated surrogate that is highly predictive of clinical benefit under treatment may fail to capture potential toxic side effects of treatment (unless toxicity and treatment efficacy are mechanistically closely related).

The often-significant time lag until the development of the ultimate clinical outcome in prospective studies presents a major challenge to validation of biomarkers as surrogate endpoints. ESRD in diabetic kidney disease, for example, may not develop for 20–30 years after the onset of diabetes. Despite this challenge, the more distant a valid surrogate biomarker is from an irreversible outcome, the more useful it is likely to be with respect to potential therapeutic interventions. The fact that the relationship between the surrogate endpoint and the hard clinical endpoint may differ between treatment types [19] suggests that it may not be practically possible to avoid lengthy studies, be they validation studies or simple clinical trials. A novel alternative to long prospective studies is based on retrospective “prediction” from archival material. Messenger RNA for gene expression studies may be obtained from formalin-fixed biopsy material [20], opening the possibility of retrospective testing of expression profiles in biopsies of patients in whom long-term follow-up is already available [21]. Use of archived serum samples has been well described in longitudinal studies such as the Framingham Heart Study. Proper processing and storage conditions for such archived specimens (particularly urine specimens) will be crucial to their effective use in retrospective studies.

In addition to purely statistical aspects of validation, issues of bias (group differences due to differences in specimen acquisition or handling unrelated to disease status), confounding, and generalizability must also be considered in biomarker validation [11]. In particular, if studies of the urinary proteome are not carefully controlled for the effects on the proteome of diurnal, gender-or age-related factors, or the influence of intercurrent illness, apparent group differences may reflect these factors rather than the disease process of interest.

### Bioinformatic and statistical methods

Statistical methods are used in biomarker discovery and validation with two basic aims: data classification and development or validation of predictive models. A classification problem generally consists of optimally partitioning a large set of individual data into a small number of classes based on some algorithm for maximizing the similarity of objects within the various classes by minimizing the composite “distance” (defined according to a specific algorithm) among members of these classes. Hierarchical clustering of gene expression array data is an example of this type of partitioning. The hierarchies are based on clusters of nested classes. Expression profiles are clustered based on their pair-wise closeness in distance. In this case, the measure of distance may be based on a form of the Pearson correlation coefficient or on a Euclidean distance between the vector representations of gene expression arrays. The particular concept of distance used, as well as the analytical methods to identify the clusters, may differ in studies of the urinary proteome when compared with studies of gene expression arrays. As many, possibly unfamiliar, statistical techniques are used in biomarker discovery and validation, a brief overview of a few of them is provided below.

**Fig. 3:**
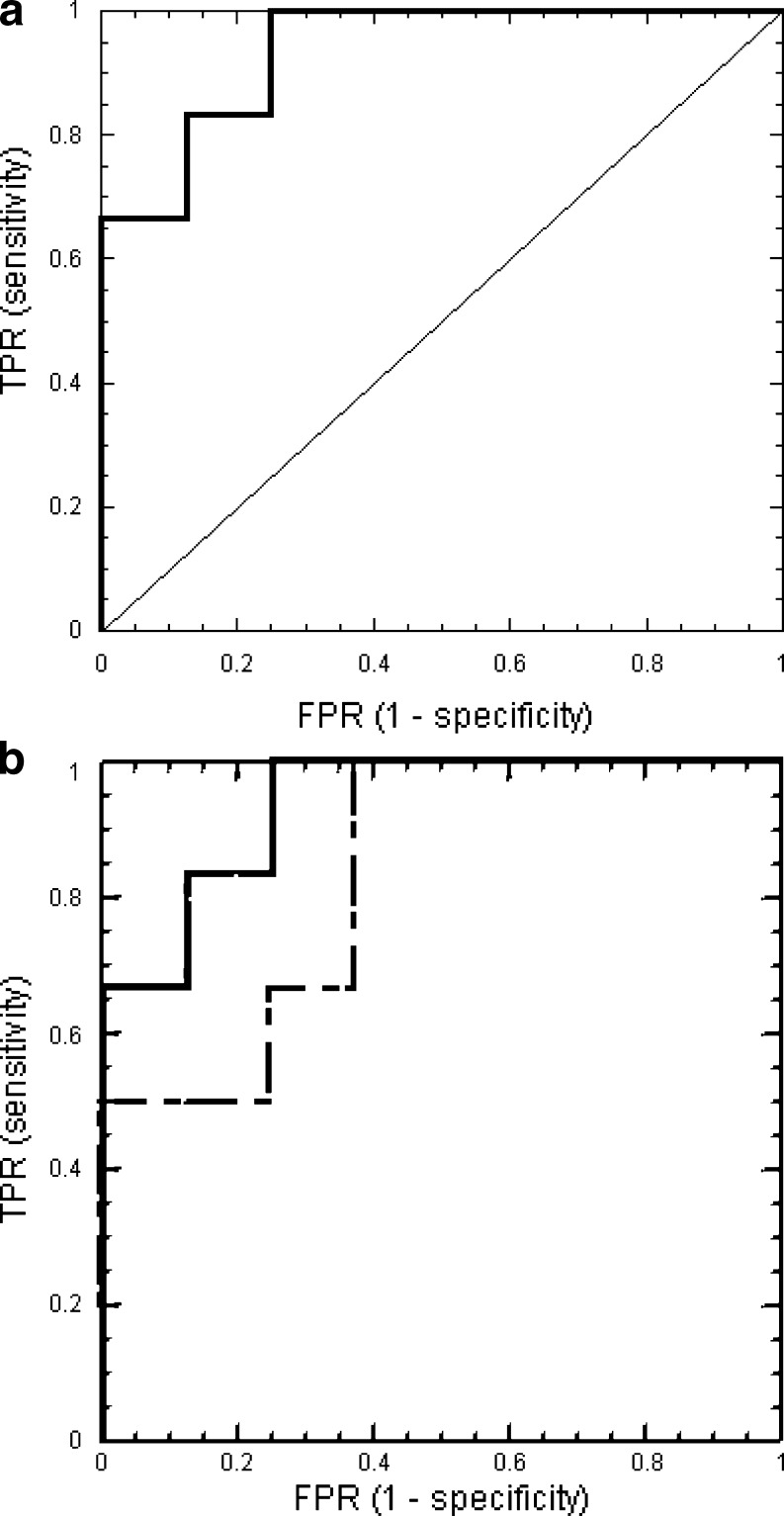
**a** Receiver operating characteristic (ROC) curve for a diagnostic test. The true positive rate (TPR) is plotted against the false positive rate (FPR). Cutoff values of the diagnostic marker that result in increasing TPR generally lead also to more false positive results. A test with no discriminatory power would follow the 45° line. **b** Comparison of two diagnostic tests using ROC curves. The curve of the better test rises more steeply in the low FPR region. The area under the curve (AUC) of the more powerful test (*solid line*) is 0.9375; that of the less powerful test (*dashed line*) is 0.8333. The AUC values are not significantly different (*P* = 0.22)

The classic statistical method used for developing predictive models relating two variables (e.g., biomarker and clinical outcome) is linear regression. Cox (proportional hazards) regression is a linear fit of a predictor variable to the log of the hazard ratio. It is often used for evaluating effects of variables on the time to arrive at a dichotomous outcome (e.g., ESRD). Receiver operating characteristic (ROC) curves are plots of the true positive rate (sensitivity) against the false positive rate (1-specificity) for a diagnostic test. ROC curves provide an optimal display of the trade-off between specificity and sensitivity for different cutoff values of a test variable. A diagnostic marker with no discriminatory power would follow the 45° line. A powerful diagnostic test has an ROC curve that rises rapidly in the low range of false positive rates (Fig. 3). The performances of different predictor variables may be compared by comparing the area under individual ROC curves (AUC) with the greater area corresponding to the more powerful test. An AUC of 0.5 corresponds to the 45° line, i.e., a test without any discriminatory power; a value of 1.0 corresponds to a perfectly predictive test. Although an ROC curve is based on values of a single predictor variable, that variable may itself be a composite of other variables, e.g., one determined by the logistic regression function on a multivariate Cox regression.

**Fig. 4:**
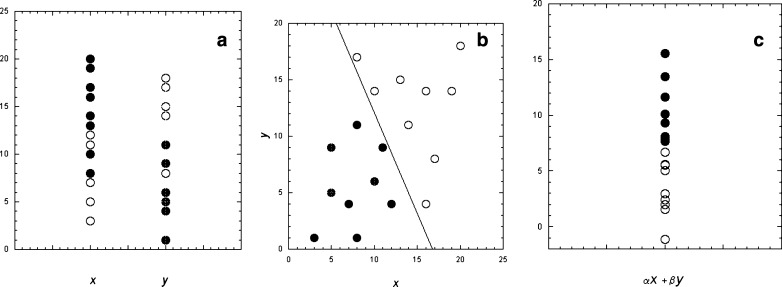
**a** Partial separation of patient groups 1 (filled circles) and 2 (open circles) based on biomarkers *x* and *y* considered individually. **b** With *y* plotted against *x*, the groups are clearly separated. *Line* represents a line of separation between the groups based on linear discriminant analysis (redrawn from [1]). **c** Transformation of the *x* and *y* variables into a composite (α*x* + β*y*) variable using the linear discriminant function preserves the separation of the two-dimensional (2D) plot while reducing the dimensionality

Multiple input variables may also be analyzed by standard multilinear regression or multivariate Cox regression. Least angle regression [22] is a recent parsimonious variant on linear regression that is less susceptible to problems of overfitting, as it “charges a price” for adding more variables. Linear discriminant analysis (LDA) finds linear combinations of quantitative features (variables) that separate a collection of individuals into classes—the classification problem again. An example of the use of two-class LDA would be to predict individuals with CKD that will progress over a given time period based on a number of features (biomarker values) measured at presentation (Fig. 4). The biomarkers considered separately may be inadequate to separate the groups (Fig. 4a), whereas the relationship between the markers (Fig. 4b) may lead to a clear distinction. A single index created from the linear discriminant function (Fig. 4c) represents the relationship among the various features that separates the classes (e.g., progressors and nonprogressors). This function reduces the dimensionality of the several features to a single value. The appropriate linear combination is estimated using a “training set” of data for which the (true) class membership is known. The linear discriminant function developed from the training data can then be used to predict class membership for subsequent individuals. Small samples with high numbers of variables can lead to instability in the estimation procedure; however, there are procedures (Friedman’s regularized discriminant analysis) that can minimize bias and misclassification risk introduced by such data imbalances.

Principal components analysis (PCA) may be seen as an alternative to LDA. Rather than separating data sets into classes, this unsupervised method is often used for reducing dimensionality of complex sets of variables while preserving a maximum of the original information content. The idea behind PCA is superficially similar to LDA: to find a new set of axes (as a linear combination of the original axes) such that the first principal component (first axis in the new set) captures the greatest amount of the total variation in the data. In the case of PCA, however, the only allowable axis manipulations are pure rotations of the axes of normalized variables (without differential weighting of the variables as in LDA). PCA essentially collapses redundant dimensions in which variables show high degrees of colinearity to other variables. Subsequent perpendicular axes (principal components) are added in order to capture the maximum amount of residual (unexplained) variation. Although this approach optimally compacts the information content of a large number of variables, a natural interpretation of the input variables may be lost in the process. A recent version of PCA considers the gene expression array variables that contribute most strongly to a composite axis as forming a family that may reflect a co-regulated gene network [23]. Many more methods exist for biomarker discovery and validation, but their description is beyond the scope of this review.

The most rigorous way to validate a predictive biomarker is to test it in an entirely new set of subjects independently sampled from the population of interest. Numerous statistical methods may be used to cross validate candidate biomarkers as predictors of hard clinical outcomes from a single sample in order to decrease the risk of model overfitting. Overfitting is particularly problematic when predictive models are developed from data derived using shotgun approaches, that is, when there are many more predictor variables (thousands) than there are outcomes (dozens to hundreds). Generally the most straightforward method of cross-validation is to hold out some of the data (traditionally about one third of the total) for use as a validation set while the rest of the data (the training set) is used in initial model development. If chosen appropriately, the validation set should give a good estimate of model performance in the general population (generalization error). The performance of the predictive model can depend heavily on patient selection for the training set [24]. Various methods exist for systematically validating a model using only a single set of data: k-fold and leave-one-out cross validation; and the bootstrap method, which differs from the other methods by drawing its subsamples with replacement from the entire sample set [25]. These methods allow the use of all of the data for model development while still permitting some independent measures of model validation. Systematic cross-validation can be used in model selection (by minimizing estimated generalization error).

### Translation from lab bench to clinical laboratory

After validation of one or more biomarkers in clinical trials, clinical application of the biomarker(s) depends on converting measurement of the biomarker into a practical clinical laboratory technique. For example, if two-dimensional (2D) gel electrophoresis on urine shows patterns in which some protein “spots” differ strongly between patient and control groups, and if these proteins are identified (by techniques such as liquid chromatography-tandem mass spectrometry (LC-MS/MS), matrix-assisted laser desorption/ionization time-of-flight (MALDI-TOF) mass spectrometry, etc. [26]), then they can subsequently be assayed quantitatively using more convenient techniques such as enzyme-linked immunosorbent assay (ELISA) or nephelometry [2]. Normal ranges and optimal diagnostic cutoff values need to be (re)established using clinical laboratory methods before widespread clinical use can begin. Gene expression patterns derived from microarray experiments may lead to polymerase chain reaction (PCR) methods for quantifying a small number of mRNA species identified in the shotgun studies as relevant to disease processes [27]. It is also necessary to harden the clinical assays (assure uniformity and reproducibility), set detectability limits, and develop standards for specimen storage and handling [1].

### Traditional univariate biomarkers in CKD

CKD has both functional and structural aspects, leading to the possibility of distinct classes of functional and structural biomarkers. Among the functional biomarkers of CKD, the glomerular filtration rate (GFR) is clearly the gold standard. Although GFR represents a single function of the kidney (the rate of formation of glomerular ultrafiltrate), it correlates strongly with a large number of unrelated kidney functions. True GFR, determined from timed urinary inulin/iothalamate clearances, is a sensitive metric of overall kidney function that can detect small changes in function over relatively short time periods [28]. It is, however, cumbersome to perform and, because of adaptation of remnant nephrons, may not indicate functional losses until significant irreversible injury (the “slippery slope”) is attained. There is also a wide range of normal values of GFR, so serial measurements may be required to establish renal function abnormality or CKD progression in its early stages. The standard clinical indicator of GFR, the serum creatinine concentration, is insensitive to changes in GFR within the range of normal or slightly impaired function [29]. Whether or not estimation equations can “salvage” the serum creatinine across a wide range of values is still subject to debate. Similarly, the utility of serum cystatin C in estimating GFR seems not to be much better than that of serum creatinine, especially in children [30]. Recent work suggests that serum levels of neutrophil gelatinase-associated lipocalin (NGAL) may correlate as well as cystatin C with GFR [31].

With regard to structural biomarkers, interstitial changes on renal biopsy, such as tubulointerstitial fibrosis or postglomerular capillary rarefaction, have been proposed to have the best association with CKD progression [32]. These parameters have been assessed at best only semiquantitatively in most studies. In addition, biopsy is an invasive procedure, and neither interstitial fibrosis nor capillary rarefaction directly reflect the fundamental structural event of CKD, i.e. nephron loss. The incidence of global glomerular sclerosis also only reflects nephron loss indirectly. Because sclerotic glomeruli are ultimately reabsorbed, the percentage of glomeruli with global sclerosis will generally underestimate the percentage of nephrons lost. The incidence of global sclerosis is also subject to significant estimation errors from biopsy specimens, although it is probably easier to estimate than absolute glomerular number [33]. Neither the percentage of sclerotic glomeruli nor the glomerular number reflect the contribution of atubular glomeruli to renal dysfunction, a factor that may be considerable [34].

Biomarkers of progression in CKD are often highly correlated with each other: GFR decreases with an increasing percentage of sclerotic glomeruli; fractional interstitial area (the quantitative index of interstitial fibrosis) is also positively correlated with the percentage of sclerotic glomeruli. Use of a composite index of these markers may help to “smooth out” some of the above-described measurement incongruities. It would, however, only compound the problems of convenience and complexity.

### Examples of classical biomarkers in CKD and their recent evolution

#### Proteinuria

Quantitative proteinuria (or albuminuria) has been the classical biomarker reflecting renal injury and predicting progression in CKD for many years. The prognostic utility of proteinuria was first demonstrated with the finding that microalbuminuria predicts with some sensitivity the subsequent development of overt nephropathy in type 1 diabetes [35, 36]. In multivariate predictive models of nondiabetic CKD progression, proteinuria has often been the strongest predictive variable [37, 38]. The use of urinary albumin excretion as a biomarker for progression risk in CKD has in fact already been endorsed by a committee constituted by the NIDDK/National Kidney Foundation (NKF) [4]. Nevertheless, proteinuria has clear limitations as a biomarker. First, it is a hybrid marker, which may reflect either acute (reversible) or chronic (irreversible) injury or in many cases a combination of both. As with GFR, the proper interpretation of proteinuria requires attention to the clinical context and may require serial determinations. For example, among diabetic subjects, even sustained microalbuminuria may remit with time: about one third of pediatric type 1 diabetic subjects with established microalbuminuria revert to normoalbuminuria after 3–6 years of follow-up [39, 40]. A similar phenomenon may be seen in Pima Indians of southern Arizona with type 2 diabetes and early renal injury [41].

The biomarker quantitative proteinuria continues to evolve from its classical version as a timed urinary excretion of total protein (or albumin). Although total urinary protein excretion may not distinguish acute from chronic renal injury, increased excretion of tubular proteins (such as β-2-microglobulin or retinol-binding protein) more likely reflects chronic injury characterized by interstitial fibrosis [42, 43]. In addition, electrophoretic patterns of urine proteins have been used to predict outcomes in CKD. A predominantly low molecular weight (LMW) pattern of urinary proteins on sodium dodecyl sulphate polyacrylamide gel electrophoresis (SDS-PAGE) predicts progression in IgA nephropathy [44], and changes in protein selectivity may parallel patterns of response to therapy in that disease [45]. The protein selectivity index (SI), which dates back to the 1960s, can be highly predictive of steroid responsiveness in idiopathic nephrotic syndrome [46]. The original formulation of the SI was as the slope of the logarithm of the fractional urinary clearances of five proteins of varying sizes (relative to transferrin) plotted against the logarithm of their molecular weights. More recently, the ratio of the fractional urinary clearance of IgG to that of either albumin or transferrin is used. The relationship between urinary albumin excretion and urinary retinol-binding protein (or β-2-microglobulin) excretion has been reported to differentiate any of a number of tubular or tubulointerstitial diseases from glomerular disease [43].

Shotgun methods applied to urine proteins (such as 2D gel electrophoresis [2, 10] or tryptic digestion followed by LC-MS/MS analysis [47]) have not as yet been used to provide potential surrogate markers of clinical outcomes in CKD despite the fact that the predictive power of LMW patterns of urinary protein suggests that this should be a productive approach. Park and colleagues [48] have described a urinary proteomic map in IgA nephropathy in which 216 protein spots were differentially present (compared with control). Fifty-nine of these proteins were identified from tryptic digests of excised spots using MALDI-TOF mass spectroscopy. Patterns of urine proteins on 2D gel electrophoresis have also recently shown promise in being able to distinguish different (pathologically defined) classes of lupus nephritis as well as predicting acuity and chronicity scores with some accuracy [49], suggesting that this technique might be used to monitor therapy. Two-dimensional differential in-gel electrophoresis (2D-DIGE) has recently been used to detect and identify differential urinary excretion of specific proteins in diabetic nephropathy [26].

The urinary excretion of novel proteins has been proposed as a biomarker, such as nephranuria as a sign of podocyte injury in diabetic nephropathy [50]. Urinary exosomes (vesicles deriving from multivesicular bodies in tubule-cell cytoplasm and exocytosed into the tubular lumen) may eventually be exploited to probe the tubule compartment of the kidney [51]. Another class of refinements for prediction is based on the temporal aspects of proteinuria, including assessing the time-average of proteinuria over 6 months [52] or the change in proteinuria after starting ACE inhibitor therapy, as a predictor of progression [53].

Urine will continue to enjoy a favored position as a source of biomarkers in CKD given its ready availability by noninvasive means and the intimate connection of urine proteins to kidney processes such as permselective ultrafiltration and tubular reabsorption. Urine may also be a simpler object of study than serum for unsupervised proteomic approaches. Interpreting the pathologic significance of proteinuria, however, remains a challenge. Proteinuria has been interpreted variously as a sign of podocyte injury or loss or as a causal link in the development of tubulointerstitial fibrosis. The pathophysiologic substrates of states of high and low proteinuria selectivity are as yet virtually completely unexplored. The only generally accepted mechanistic interpretation is that high molecular weight proteinuria is considered to reflect decreased glomerular permselectivity, whereas LMW proteinuria is thought to be due to defects in tubular uptake of filtered proteins [54].

#### Cyturia

Seventy years ago, Thomas Addis assessed kidney injury by determining the quantitative rates of urinary red and white cell excretion (as well as quantitative proteinuria). Almost 10 years ago, Masanori Hara and colleagues [55] described the presence of podocytes in the urinary sediment of children with a variety of glomerular diseases. Recently, it has been shown that podocytes are shed into the urine in varying amounts in both health and disease, that podocyturia may be quantified as a ratio to urinary creatinine, and that this ratio differs between active and quiescent disease [56]. The appearance of podocytes in the urine is a mechanistically appealing index of glomerular injury inasmuch as podocytes are considered to be postmitotic cells that contribute importantly to glomerular tuft stability [57]. Their appearance in the urine may thus be expected to correlate with their loss from the glomerulus, although this has not yet been definitively demonstrated. Decreased glomerular podocyte number (podocytopenia) has been related to the development of glomerular sclerosis and loss of kidney function in both humans and experimental models [58–60]. Nephrin and podocin mRNA excretion rates have been correlated with the subsequent loss of renal function [61]. These species may prove to be more easily measured and convenient surrogates of podocyturia, although some caution is indicated in extrapolating urinary mRNA excretion to podocyte excretion due to the possible influence of podocyte cell fragments in the urinary sediment [62]. No study to date has shown a correlation of directly measured podocyturia with urinary mRNA excretion. Although the cells of origin cannot be determined, urinary cytokine mRNA excretion has recently been proposed to be able to distinguish class IV lupus nephritis from other classes better than standard clinical markers, such as erythrocyturia or proteinuria [63].

### The challenge of multiplex biomarkers

The discriminatory power of biomarkers may be enhanced when multiple biomarkers are used together (Fig. 4). Despite this, predominantly univariate candidates have been studied as potential novel biomarkers in CKD [urinary cytokines such as connective tissue growth factor (CTGF) or transforming growth factor β (TGFβ); podocyte or mononuclear cell excretion; urinary β-2-microglobulin/creatinine or retinol binding protein (RBP)/albumin ratios)]. Even shotgun approaches such as 2D-DIGE on urine proteins or gene expression arrays have usually been mined for individual (or small numbers of) candidates. Part of the problem is that the desired output has been a one-dimensional (1D) construct called kidney dysfunction. This is a natural result of our reductionist thought processes. This is probably why techniques that promise dimensionality reduction are so appealing. Even when a complex pattern (e.g., of urine proteins) is correlated with a clinical outcome, it is invariably hard to develop an integrative paradigm that assigns a pathophysiologic meaning to the pattern. It remains a challenge to visualize a multiplex biomarker other than as a series of 1D cuts through the data.

A multiplex panel of biomarkers will, however, almost certainly give a more comprehensive and robust view of the multidimensional pathologic processes of CKD and better reflect the totality of treatment effects, including toxicity. Such a panel can be overlaid on an equally rich panel of genetic and environmental susceptibility factors to maximize its utility. The effective use of multiplex biomarkers in therapeutic decision making will be a lynchpin in the development of individualized medicine. We need to develop new visualization and interpretation techniques that will allow us to maintain a maximum of dimensionality in our uses of biomarkers.

## Questions (Answers appear following the reference list)

1. What is likely to be the most challenging step in the development of biomarkers from gene expression array data?
  a. Discovery
  b. Validation
  c. Assay hardening
  d. Developing standards for sample handling
2. Which method is *not* a common pathway to discovery of novel biomarkers?
  a. Shotgun methods (e.g., expression microarrays)
  b Pathomechanism-driven research
  c. Randomized controlled drug trials
  d. Studies on animal models
3. An ideal biomarker should be all of the following *except*:
  a. Quantitative
  b. Noninvasive
  c. Necessary for FDA drug approval
  d. Reproducibly measurable
4. Examples of biomarkers include all of the following *except*:
  a. Urine podocyte/creatinine ratio
  b. Renal pelvic diameter on prenatal ultrasound
  c. Blood pressure “burden” in a 24-h ambulatory record
  d. Angiotensin converting enzyme (ACE) I/D gene polymorphism
5. Surrogate endpoint biomarkers
  a. May be arrived at by careful physiologic reasoning
  b. Always reflect both the efficacy and toxicity of treatments
  c. May be used to shorten clinical trials
  d. Are usually unrelated to hard clinical endpoints
6. A receiver operating characteristic (ROC) curve
  a. Is a plot of the true positive versus the false positive rate of a test
  b. Cannot be used to compare two different diagnostic tests
  c. Is a plot of sensitivity versus specificity
  d. Is most useful when it follows the 45° line (line of identity)
7. Cross-validation of a predictive model
  a. Consists of checking one model against a different model
  b. May consist of checking a model developed on a training set for accuracy when applied to an independent validation set
  c. Provides no protection against model overfitting
  d. Is best done using ROC curves

Answers to Questions

1. B

2. C

3. C

4. D

5. C

6. A

7. B
